# Second Generation Drug-Eluting Stents for Endovascular Treatment of Ostial Vertebral Artery Stenosis: A Single Center Experience

**DOI:** 10.3389/fneur.2019.00746

**Published:** 2019-07-16

**Authors:** Santiago Ortega-Gutierrez, Gloria V. Lopez, Randall C. Edgell, Aldo A. Mendez, Sudeepta Dandapat, Jorge A. Roa, Cynthia B. Zevallos, Andrea L. Holcombe, David Hasan, Colin P. Derdeyn, James Rossen, Edgar A. Samaniego

**Affiliations:** ^1^Department of Neurology, University of Iowa Hospitals and Clinics, Iowa City, IA, United States; ^2^Department of Neurosurgery, University of Iowa Hospitals and Clinics, Iowa City, IA, United States; ^3^Department of Radiology, University of Iowa Hospitals and Clinics, Iowa City, IA, United States; ^4^Department of Neurology, SSM Health Saint Louis University Hospital, St. Louis, MO, United States; ^5^Department of Internal Medicine - Cardiovascular Medicine, University of Iowa Hospitals and Clinics, Iowa City, IA, United States

**Keywords:** vertebral artery stenosis, extracranial atherosclerotic disease, stenting, drug eluting stent, restenosis

## Abstract

**Objective:** To report a single-center experience using drug-eluting balloon mounted stents (DES) for endovascular treatment of atherosclerotic ostial vertebral artery stenosis (OVAS).

**Background:** Posterior circulation is affected in up to 25% of strokes, 20% of them resulting from atherosclerotic OVAS. The optimal management of symptomatic OVAS remains controversial. DES have been introduced to improve restenosis rates.

**Methods:** We retrospectively analyzed prospectively collected data from patients with dominant OVAS who underwent endovascular treatment with second-generation DES placement. Patient demographics, clinical presentation, comorbidities, stenosis severity, stent features, technical success, complications, and imaging follow-up were assessed.

**Results:** Thirty patients were treated, predominantly male (86.6%). Sixteen patients presented with an acute stroke or TIA and fourteen were treated on an elective basis due to symptomatic chronic stenosis and contralateral occlusion. Comorbidities included hyperlipidemia (83%), hypertension (70%) and prior stroke (63.3%). Mean ostial stenosis at presentation was 80 ± 14.8%. Twenty-one patients had contralateral VA involvement. DES deployment was technically successful in all patients using everolimus eluting stents in 30 lesions and zotarolimus eluting stents in two. One technical complication (stent migration) and three (10%) minor peri-procedural complications occurred. Complications included one asymptomatic ischemic infarct in the posterior circulation, one femoral artery thrombosis and one post-procedure altered mental status secondary to contrast induced neurotoxicity. Mean imaging follow-up was 8.8 months. Two (7.6%) patients had in-stent restenosis and underwent retreatment with angioplasty. There were no procedure-related mortalities.

**Conclusion:** Our study confirms the feasibility of deploying DES for the treatment of ostial vertebral artery stenosis with low peri-procedural risk and low medium-term rates of re-stenosis.

## Introduction

Posterior circulation strokes represent ~25% of all strokes. Of those, up to 20% result from atherosclerotic ostial vertebral stenosis (OVAS) (1, 2). Although the optimal management of OVAS has not been well-defined, there is good clinical consensus that the initial management should include medical therapy (MT) with antiplatelet agents and cardiovascular risk factors control. The persistence of symptoms despite optimal medical treatment, leads to the consideration of either surgical or endovascular options. Although low stroke (1.9%) and death (0.6%) rates have been reported with the use of open surgical bypass or endarterectomy for extracranial artery disease (3), significant complication rates approaching 20% were described when specifically applied for OVAS (3, 4).

Endovascular treatment has shown to be a safer and less invasive alternative to open surgery. The vertebral artery stenting (VAS) periprocedural complication rate ranges from 1.9 to 2.96% (5). However, a high restenosis rate has been observed with OVAS when bare metal stents (BMS) are used (6). Anti-proliferative drug-eluting stents (DES) for treatment of coronary vascular disease has led to reduced rates of restenosis in this vascular bed (7, 8), leading neurointerventionalists to adopt this technology in the treatment for OVAS. DES provide local, controlled release of antiproliferative agents targeting the suppression of neointimal hyperplasia seen in in-stent restenosis (ISR) (9). First-generation DES, such as sirolimus and paclitaxel-eluting stents, have shown superior results when compared with BMS (bare metal stents) in some case series, with no improvement in others (10, 11). Debate continues on their safety and efficacy given their association with delayed vascular healing and late stent thrombosis, especially after discontinuation of dual antiplatelet therapy (7). Thinner and more biocompatible second-generation DES have been designed to improve efficacy, safety, and device performance.

These newer devices have proven to be clinically superior to early DES in the treatment of coronary vascular disease (8, 12). Additionally, the percentage and rate of in-stent restenosis has been demonstrated to be lower with DES. With the use of BMS restenosis becomes clinically evident as early as 6–12 months following intervention (13). In contrast with restenosis observed in first and second-generation DES which generally occurs later in time (13–16%, 5–6.3% at 5 years, respectively) (14, 15).

We report our initial experience using second-generation DES for the treatment of symptomatic OVAS. We aim to evaluate the procedural complications and clinical and radiographic outcome. To our knowledge, this is first large, single-center series treating OVAS with second-generation DES to be reported in the literature.

## Matherials and Methods

### Patient Population

This is a retrospective analysis of prospectively collected data from patients presenting from January 2017 to September 2018 in our comprehensive stroke center. Patients were included if they experienced: (1) stroke or transient ischemic attacks (TIA) in the posterior circulation despite maximal MT or (2) severe stenosis (>70%) based on initial computerized tomography angiography (CTA) of a dominant or co-dominant vertebral artery ostium. All patients were independently evaluated clinically by a stroke neurologist before their referral for possible revascularization procedures. Stroke was defined as positive diffusion-weighted (DWI) in the acute phase or fluid-attenuation inversion recovery (FLAIR) in the subacute-chronic phase in the posterior circulation. TIA was based on a stroke physician's neurological assessment without DWI neurologic findings. Maximal MT included single or dual antiplatelet and high dose statin.

All vascular images were independently reviewed by a stroke physician and a fellowship-trained neurointerventionalist (SOG, EAS) and included in the study after consensus was met. CTA was used to evaluate the degree of stenosis using adjusted North American Symptomatic Carotid Endarterectomy Trial (NASCET) criteria to the posterior circulation (16). Briefly, the diameter of the vertebral artery (VA) was measured on images that showed best arterial opacification in the thin Maximum Intensity Projection (MIP) series. The narrowest point of the VA ostium was measured (D stenosis). Normal reference vessel diameter was measured distally to the affected ostium at a non-diseased, non-tortuous segment of the cervical VA in which the walls of the vessel seemed parallel and the vessel diameter was consistent for at least one centimeter (D normal). The degree of stenosis (%) was then quantified as [1 – (D stenosis/D normal)] ×100, where D stenosis = diameter of the artery at the site of the most severe stenosis, and D normal = diameter of the proximal normal artery. Patients' demographic profile, comorbidities and risk factors and clinical presentation were collected from our electronic medical record system. The study was conducted in accordance with our local institutional review board (IRB) regulations.

### Stent Placement Protocol

All patients undergoing endovascular stenting as an elective procedure were administered dual antiplatelet therapy consisting of 325 mg aspirin and 75 mg clopidogrel daily (Plavix, Bristol-Myers Squibb/Sanofi Pharmaceuticals, New York, New York) 5–7 days before treatment. Acute, symptomatic patients were loaded prior to the procedure with 325 mg of aspirin and 300 mg of clopidogrel. Heparinization during the procedure was standard, with the goal of an activated coagulation time >250 s before any attempt to cross the region of stenosis. The route of arterial access (i.e., transfemoral, transradial) was determined by the neurointerventionalist. With the guide catheter located in the subclavian artery near the VA ostium, an angiographic roadmap was used to cross the lesion. At this point, the VA ostium was carefully traversed with a 200 cm [0.014-in] microwire. In high-grade stenosis cases, an undersized, semi-compliant coronary angioplasty balloon (Emerge, Boston Scientific Corporation, Natick, MA) was inflated in the VA ostium before stent delivery. Distal protection devices were not used in any of the patients. A balloon-expandable rapid exchange everolimus (XIENCE Alpine, Abbott Laboratories, Abbott Park, IL) or zotarolimus (Resolute Integrity, Medtronic, Minneapolis, MN) eluting coronary stent was then deployed across the stenosis, attempting to completely cover the VA ostium with minimal protrusion into the subclavian artery at the proximal side of the ostium. Immediate follow-up angiography was done, and post-stent angioplasty was performed when needed to achieve residual luminal stenosis of <20%. At the end of the procedure, femoral arterial access sites were secured with a closure device (Angioseal, St. Jude Medical, St. Paul, MN). All procedures were performed using conscious sedation for continuous patient neurological monitoring. Patients were admitted to the stroke unit (NICU) for overnight monitoring. Patients were continued on dual antiplatelet medications for a minimum of 12 months after treatment and on aspirin indefinitely.

### Clinical and Radiographic Outcomes

The National Institutes of Health Stroke Scale (NIHSS) was calculated at admission and discharge in all patients. Technical aspects of the procedure including, complications, radiological, and clinical outcomes were recorded and analyzed. All complications were noted and reported during the time of hospitalization. Complications were defined as follows: (a) “major” adverse event was a new neurological deficit or worsening of the pre-existing deficit measured by an increase of NIHSS >4 points after the procedure and lasted longer than 24 h; (b) “minor” adverse events were considered as events that resolved within 24 h with no clinical sequelae; (c) “technical” as any issues encountered by the proceduralist during the deployment of the stent. A modified Rankin Scale (mRS) was assessed at discharge and at the 3-month follow up visit.

Percent stenosis was re-calculated for each angiographic study (at presentation, immediately after stenting and at 6–12 month follow-up) and categorized into four groups: insignificant (0–25%), mild (26–50%), moderate (51–75%), and severe (76–100%). ISR was defined as angiographic evidence of >50% stenosis at last available follow-up. Stent failure was defined as ISR cases in which symptomatic status of the patient warranted target-lesion revascularization and re-retreatment.

## Results

### Patient Characteristics

Thirty patients were identified. Most patients were male (86.6%) with a median patient age of 69 years (IQR = 58–77). The most commonly reported comorbidity was hyperlipidemia (83.3%) followed by hypertension (70%). Ten patients (33.3%) presented with acute TIA symptoms and six (20%) were admitted due to stroke (Table 1). Among the symptomatic patients, the mean NIHSS score at admission was 3.7 ± 7.4 points and 1.8 ± 3.6 at discharge. The mean mRS at 90-day follow up for acutely hospitalized patients was 3.4 ± 0.66 and 0.85 ± 0.79 for patients treated on an elective basis. Due to late presentation, none of the patients who suffered stroke received thrombolytic treatment.

**Table 1:** Baseline characteristics.

	***N* (%)**
**Demographics**	
Age ± IQR (*N* = 30)	69 ± 19.25
Gender–male	26 (86.6)
Ethnicity	
White	26 (78)
African American	2 (6.6)
Other	2 (6.6)
**Symptoms**	
Acute stroke	6 (20)
Acute transient ischemic attack	10 (33.3)
Subacute or chronic presentation	14 (46.6)
**Risk factors**	
Hyperlipidemia	25 (83.3)
Essential hypertension	21 (70)
Previous stroke	19 (63.3)
Smoking	13 (40)
Coronary artery disease	10 (33.3)
Diabetes mellitus	8 (26.6)
Cancer	5 (16.6)
Peripheral vascular disease	5 (16.6)
Atrial fibrillation	5 (16.6)
Neck radiation	4 (13.3)
Drug abuse	2 (6.6)
**Coexisting arterial disease**	*N* = 32
Unilateral ICA	5 (15.6)
Bilateral ICA	6 (18.7)
Contralateral VA	21 (65)
Occluded	10 (47.7)
Stenosis >50%	5 (23.8)
Hypoplastic or ending in PICA	6 (28)

*IQR, Interquartile range; ICA, internal carotid artery; VA, vertebral artery; PICA, posterior inferior cerebellar artery*.

**Figure 1:**
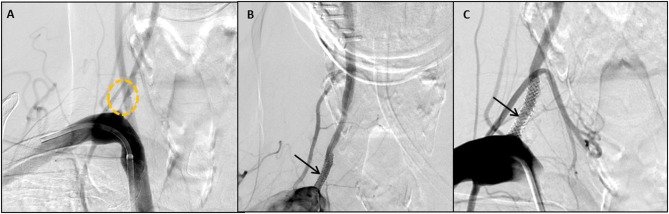
Case No 8 **(A–C)**. **(A)** Pre-stenting posteroanterior vertebral artery injection, showing a right vertebral artery origin stenosis (circle). **(B)** Immediate post-stent placement posteroanterior projection showing close apposition of the stent to the wall of the artery (arrow) **(C)** 12 month follow up vertebral artery injection showing mild in-stent restenosis of 10%.

Thirty-two stents were placed in 30 patients, with two patients receiving bilateral stents. The dominant vertebral artery was stented in all cases, most commonly on the right side (62.5%). DES deployment was technically successful in all patients without post-procedural residual stenosis (Figures 1A–C). There was no kinking of any of the stents. Immediate restoration of blood was present in all cases on immediate post-deployment angiography. There was no angiographic distal embolization or dissection in the post-cranial anterior-posterior and lateral angiograms.

There were no major complications. Three (10%) minor periprocedural complications occurred: a small cerebellar silent infarct, a femoral artery thrombosis and a patient that suffered mild encephalopathy attributed to contrast induced neurotoxicity after all other etiologies were ruled out. A technical complication of distal stent migration also occurred which required a second DES placement. An octogenarian subject died because of acute respiratory failure not related to the OVAS pathology (Table 2).

**Table 2:** Clinical presentation, device characteristics, and intra and peri-procedural events.

**Case**	**Clinical presentation**	**Stenosis %**	**Angio-plasty**	**Stent width/length (mm)**	**Stent coated drug**	**Peri-procedural complications**
1	Stroke	80	4	3.5 ×18	Everolimus	
2	None/Elective	95	2	2.75 ×12	Everolimus	
3	None/Elective	70	1	4 ×12	Everolimus	
4	Stroke	90	1	3.5 ×26	Zotarolimus	
5	TIA	60	1	4 ×26	Everolimus	
6	TIA	50	4	4 ×12	Everolimus	Proximal stent migration
7	TIA	50	1	3 ×12	Everolimus	
8	None/Elective	90	2	3 ×16	Everolimus	
9	None/Elective	90	1	4 ×12	Everolimus	
10	TIA	80	2	4 ×12	Everolimus	
11	Dissection	90	2	3.5 ×12	Everolimus	
12	Stroke	95	2	3.5 ×12	Everolimus	
13	None/Elective	90	2	3 ×12	Everolimus	
14	None/Elective	70	1	4 ×16	Everolimus	
15	None/Elective	95	2	4 ×8	Everolimus	
16	TIA	99	2	3 ×8	Everolimus	Asymptomatic minor stroke
17	TIA	90	2	4 ×12	Everolimus	
18	None/Elective	90	2	2.25 ×12	Zotarolimus	
19	Stroke	90	2	3.5 ×16	Everolimus	
20	TIA	85	1	3.5 ×8	Everolimus	
21	TIA	60	1	3.5 ×12	Everolimus	
22	TIA	90	1	4 ×8	Everolimus	
23	None/Elective	50	1	2.5 ×8	Everolimus	
24	None/Elective	70	2	4 ×12	Everolimus	
25	Stroke	60	2	4 ×8	Everolimus	
26	None/Elective	99	1	4.5 ×24	Everolimus	
27	None/Elective	80	1	4.5 ×37	Everolimus	
28	None/Elective	60	1	2.25 ×9	Everolimus	
29	Stroke	75	1	4 ×8	Everolimus	Femoral artery thrombosis
30	None/Elective	65	1	3.5 ×12	Everolimus	
31	TIA	90	1	3.25 ×15	Everolimus	
32	None/Elective	60	1	4 ×8	Everolimus	Contras induced neurotoxicity

*TIA, Transient ischemic attack*.

**Figure 2:**
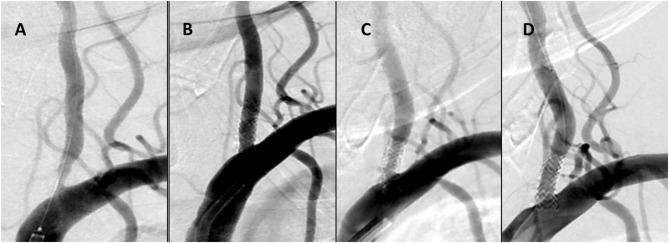
Case No 13 **(A–D). (A)** Pre-treatment vertebral artery injection showing a 80% stenosis **(B)** Immediate post-treatment injection with wide patency of the stented artery **(C)** 7 month follow up injection demonstrating a 70% ISR **(D)** Immediate re-treatment injection demonstrating ISR resolution.

Clinical and radiological outcomes are summarized in Table 3. On clinical follow-up, improvement of symptoms was observed in patients with subacute/chronic presentation and no recurrence of stroke or TIA occurred in all patient. Follow-up studies were available in 26 patients with 26 lesions. CTA and DSA was obtained in 13 (50%), CTA only in eight (30.7%), and DSA only in five patients (19.3%). The means of the longest follow ups were 9.09 months and 8.83 months for CTA and DSA, respectively (range 3–24 months). During follow-up, moderate and severe ISR occurred in two patients (7.6%) who underwent target-lesion revascularization and vessel retreatment (Figures 2A–D).

**Table 3:** Clinical and radiographic outcomes.

**Case**	**NIHSS pre/discharge**	**mRs at 3–6 m**	**Imaging F/U (months)**	**Restenosis (%)**	**Retreatment (Yes/No)**
1	28/12	6	CTA (3)	0	No
2	0/0	2	–	–	–
3	0/0	0	DSA (6)	0	No
4	0/0	0	DSA (7, 15) CTA (1,12,18)	80	Yes
5	0/0	0	CTA (3,15, 24) DSA (10)	10	No
6	0/0	1	CTA (3) DSA (7)	40	No
7	3/3	2	CTA (3)	0	No
8	0/0	0	CTA (3) DSA (12)	10	No
9	0/0	0	CTA (6) DSA (12)	0	No
10	0/0	0	CTA (3) DSA (6)	0	No
11	0/0	0	–	–	–
12	16/10	4	CTA (3,11) DSA (5)	0	No
13	0/0	3	CTA (4) DSA (6)	70	Yes
14	0/0	3	CTA (11,24) DSA (6)	30	No
15	0/0	0	CTA (3)	0	No
16	0/0	0	CTA (10,24) DSA (13)	0	No
17	0/0	0	CTA (6,18) DSA (10)	0	No
18	0/0	1	CTA (3,12)	0	No
19	0/0	2	CTA (3)	0	No
20	2/2	3	CTA (3) DSA (12)	0	No
21	0/0	0	DSA (8)	30	No
22	1/0	0	–	–	–
23	1/1	3	CTA (3,10)	No	No
24	3/3	1	DSA (6)	No	No
25	7/1	3	–	–	–
26	2/2	2	–	–	–
27	2/2	2	–	–	–
28	0/0	1	CTA (3) DSA (8)	0	No
29	4/4	1	DSA (11)	0	No
30	0/0	3	CTA (10)	0	No
31	3/0	1	DSA (6)	0	No
32	3/0	1	CTA (3)	0	No

*CTA, computed tomography angiography; DSA, digital subtraction angiography; F/U, follow-up; –, No available data*.

## Discussion

The natural history of OVAS has not been well defined. Due to the low flow and high turbulence, atherosclerotic disease commonly affects the origin and proximal portion of the vertebral artery (17). Previously, OVAS was considered a relatively benign finding in which the contralateral vertebral artery could compensate for the lack of flow. Recent data has suggested that atherosclerotic vertebral plaque act in a similar fashion to symptomatic carotid stenosis, with a high recurrent stroke risk (18). As such, treatment, whether medical or interventional, is warranted for stroke prevention. The high stroke rate (1), as well as the persistence of symptoms despite optimal medical treatment, justifies the consideration of revascularization treatment.

The evidence to support the use of VAS for the treatment of OVAS remains controversial. Randomized clinical trials comparing BMS vs. MT have shown variable results. In the Carotid and Vertebral Artery Transluminal Angioplasty Study (CAVATAS), eight patients were randomized to MT and eight patients underwent successful endovascular stenting. No strokes or death occurred within 30 days of the procedure or at follow up (mean of 4.5 years) in the vertebrobasilar territory stroke (19). The Vertebral Artery Stenting Trial (VAST) demonstrated significantly increased 30-day rates of major periprocedural complications (vascular death, myocardial infarction or stroke) in patients undergoing stenting vs. medical therapy (5 vs. 2%). However, the adverse events occurred in intracranial vertebral artery lesions, not in subjects treated for OVAS. More recently, in 2017, the Vertebral Artery Ischaemia Stenting Trial (VISIT) reported lower complication rates (fatal or non-fatal stroke in any arterial territory) within the stenting group (2%) compared to the medical group (4%) at follow-up (median 3.5 years) (20). There were no periprocedural complications with extracranial stenting group. Differences in the effect sizes of VAS using BMS could be explained, at least in part, due to the inclusion of both intracranial and extracranial stenosis patients in the three trials. As reported in the Stenting and Aggressive Medical Management for Preventing Recurrent Stroke in Intracranial Stenosis (SAMMPRIS) trial, patients with symptomatic intracranial stenosis might exhibit a significantly different 30-day rate of stroke and death when treated with intracranial self-expanding stents vs. MT (14.7 vs. 5.8%) (21). These rates, in conjunction with anatomical variance at the different vertebral segments, suggest that OVAS and intracranial stenosis are two different entities with different stroke-procedural risk and need to be studied separately.

First-generation DES for OVAS was first introduced by Ko et al. in an attempt to minimize the risk of restenosis related to the use of BMS (22). DES have anti-migratory and anti-proliferative effects on vascular smooth muscle that minimize restenosis associated with neointimal hyperplasia. Initial studies reported low rates of periprocedural (23–25) and major complications rates (0.9–5.1%) (26, 27). Two meta-analyses comparing BMS and first-generation DES have shown that BMS has a significantly higher rate of recurrent symptoms, restenosis rates, and target vessel retreatment (5, 10). The first meta-analysis demonstrated significantly higher restenosis rate for BMS (23.7 vs. 8.2%) compared to DES (10). The second meta-analysis showed a significantly higher rate of recurrent symptoms (11.26 vs. 2.76%), restenosis (33.57 vs. 15.49%), and retreatment (19.21 vs. 4.83%) when comparing BMS to DES (5).

Although first-generation DES have shown promising results in the treatment for OVAS, the coronary literature suggests they might be associated with delayed arterial healing and premature neoatherosclerosis (9). Second-generation DES have been developed to decrease the inflammatory response and achieve more rapid endothelialization. This new generation of stents is made of cobalt/platinum-chrome which provides improved radial strength and thinner struts compared to stainless steel used on the first-generation, decreasing arterial injury and restenosis risk. Furthermore, the more biocompatible polymer coating (e.g., PBMA poly-n-butyl methacrylate) reduces inflammatory response and thrombosis resulting in better physiological arterial healing and decreased risk of acute periprocedural thrombosis. A large meta-analysis of four randomized clinical trials, consisting of 6,792 patients, compared everolimus to paclitaxel-eluting stents in patients with acute coronary syndrome (9). It demonstrated significant reduction in rates of early (30 days) (0.2 vs. 0.9%), late (31–365 days; 0.2 vs. 0.6%), and very late (>365 days; 0.2 vs. 0.8%) stent thrombosis (8). Our study is the first large, single-center case series reporting the experience using second-generation DES (zotarolimus and everolimus-eluting stents) to treat symptomatic acute and chronic OVAS non-responsive to standard MT. Feasibility of the technique is evidenced by the high rate of technical success (100%). Our overall periprocedural complication rate was 13.3%, all which were classified as minor complications. There were no perioperative mortality or major complications.

In follow up imaging, only two patients (7.6%) suffered significant ISR of 50 and 80% at 6 and 15 months respectively, requiring retreatment (Figures 2C,D). Literature suggests that the most significant factors associated with ISR in patient with OVAS include cardiovascular comorbidities such as hypertension and diabetes and the length of the lesion (2, 28). In addition, the VA ostium has an increased risk of restenosis, perhaps due to its high elastin composition and the increased mechanical stress caused by hypermobility of the subclavian-vertebral artery junction (27). Both of our patients with ISR have a history of hypertension, hyperlipidemia, and lesion lengths >10 mm (29). The vessel diameters were also small, another predictor of ISR (30). Of note, one of the patients had a history of head and neck cancer and underwent palliative radiation years prior. Neither patient was symptomatic, possibly related to the development of collateral circulation.

Dual antiplatelet regimen was employed in all our patients to protect against early and late ischemic events after DES implantation. The optimal duration of dual antiplatelet therapy after implantation of DES remains controversial. Although most series describing use of DES for VA stenting have described a duration of 3–6 months of dual antiplatelet therapy (24), current American College of Cardiology/American Heart Association guidelines recommend at least 6 months of dual antiplatelet therapy, with a prolongation of therapy per physician's preference (31, 32). All patients in our study received a minimum of 12 months, with some maintained longer depending on the evidence and degree of restenosis on follow up imaging.

The main limitations of this study include its single-arm, single-center design, a lack of control groups, and the relatively small number of patients. There is also a difference in the timing of the procedure related to the onset of symptoms. Some patients presented acutely while other were treated electively. Lastly, the lack of long term angiographic follow up might introduce bias in the estimation of the true rate of restenosis.

## Conclusion

Our study represents preliminary data of safety and feasibility of using second-generation DES in treating OVAS. Technical challenges were minimal and periprocedural morbidity was low. A prospective, single-arm, large multicenter phase 2 registry represents the natural next step to better define the safety profile of these devices in the treatment of OVAS before comparing other current therapies in a randomized clinical trial.

## Data Availability

Additional unpublished angiographic and cone beam CT data for each case are available to readers of the journal. Please email all requests to santy-ortega@uiowa.edu.

## Ethics Statement

The study was approved by the institutional review board of University of Iowa Hospitals and Clinics, Iowa City, Iowa. IRB approved exemption of consent. IRB ID: 201712811.

## Author Contributions

SO-G and ES performed endovascular procedures and contributed to manuscript conception, design, creation, editing, and revision. GL, AM, and JAR contributed to manuscript conception, design, creation, editing, and revision. CZ and GL contributed to data collection. AH contributed to manuscript conception, design, editing, and revision. SD contributed to manuscript conception and design. RE, CD, JR, and DH contributed to the editing, and revision.

### Conflict of Interest Statement

The authors declare that the research was conducted in the absence of any commercial or financial relationships that could be construed as a potential conflict of interest. The reviewer WG declared a past co-authorship with several of the authors to the handling editor.
